# Age-Related Changes in Gut Health and Behavioral Biomarkers in a Beagle Dog Population

**DOI:** 10.3390/ani15020234

**Published:** 2025-01-16

**Authors:** Anna Fernández-Pinteño, Rachel Pilla, Jan Suchodolski, Emmanuelle Apper, Celina Torre, Anna Salas-Mani, Xavier Manteca

**Affiliations:** 1 Department of Research and Development, Affinity Petcare, 08902 L’Hospitalet de Llobregat, Spain; eapper@affinity-petcare.com (E.A.); ctorre@affinity-petcare.com (C.T.); asalas@affinity-petcare.com (A.S.-M.); 2 Gastrointestinal Laboratory, Department of Small Animal Clinical Sciences, Texas A&M University, College Station, TX 77843, USA; rpilla@cvm.tamu.edu (R.P.); jsuchodolski@cvm.tamu.edu (J.S.); 3 School of Veterinary Science, Universitat Autònoma de Barcelona, 08193 Bellaterra, Spain; xavier.manteca@uab.cat

**Keywords:** fecal microbiota, behavior, canine, aging, health, nutrition, gut–brain axis

## Abstract

The gut and the gut microbiome communicate with the nervous system through the gut–brain axis via neuroimmune and neuroendocrine mechanisms. Despite existing research, studies exploring this link in aging dogs are limited. This study aims to examine multiple blood and fecal biomarkers of intestinal health, along with various behavioral indicators based on saliva, blood, observations, and activity, in different age populations (junior: <2 y.o.; adult: 2–7 y.o.; senior: >7 y.o.) of thirty-seven Beagle dogs. In our study, *Bacteroides* were significantly higher in senior dogs. The relative abundance of *Faecalibacterium* and *Blautia* showed age-related trends, higher in senior and junior dogs, respectively. Fecal short-chain fatty acid concentration, especially acetate, increased with age, while propionate was higher in junior dogs. For the behavioral indicators we considered, blood thyroxine concentration, playing, exploring, and total activity were higher in junior dogs. The differences observed between the biomarkers of gut health and behavior, particularly those significant for the age correlations, emphasize the importance of considering age-related factors when studying the gut microbiome and behavior. However, further research is needed to better understand the mechanisms and specific pathways involved in the relationship between the studied biomarkers and age.

## Simple Summary

The gut and its microbiome communicate with the brain through the gut–brain axis. The examination of the relationship between the gut and the brain concerning aging is particularly relevant for preserving the quality of life in senior dogs. Studies investigating this axis in dogs of different age groups remain limited. Thus, this study aims to examine multiple blood and fecal biomarkers of intestinal health, along with various behavioral indicators based on saliva, blood, observations, and activity, in a different age population (junior: <2 y.o.; adult: 2–7 y.o.; senior: >7 y.o.) of thirty-seven Beagle dogs. The results showed that *Bacteroides* were significantly higher in senior dogs. The relative abundance of *Faecalibacterium* and *Blautia* showed age-related trends, higher in seniors and juniors, respectively. Fecal short-chain fatty acid concentration, especially acetate, increased with age, while propionate was higher in junior dogs. For the behavioral indicators we considered, blood thyroxine concentration, playing, exploring, and total activity were higher in junior dogs. These findings suggest that the relationship between gut health and behavior varies with age. This highlights the importance of taking age into account when studying gut health and behavior. However, more research is needed to fully understand the mechanisms behind these age-related changes.

## 1. Introduction

The gut microbiome is composed of bacteria, archaea, fungi, protozoa, and viruses inhabiting the gastrointestinal tract. These communities play a crucial role in important metabolic functions and essential immune responses that protect against pathogens and help maintain the well-being of their host, contributing to an equilibrated health status in dogs and cats [1,2].

There is increasing scientific evidence supporting the relationship between the gut microbiota and different organs and systems. Multiple studies in mammals have demonstrated that changes in the gut microbiota are associated with some pathological states, for example, inflammation, obesity, metabolic diseases [1], changes in behavior [2], and emotional states [3]. The pathways through which one body part biochemically communicates with another body part are known by the scientific community as the ‘axis’. The gut and the gut microbiota participate in specific axes through the portal vein, the nerve pathways, or through the intestinal barrier into the blood circulation. In the gut–brain axis (GBA), the gut microbiota communicates to the host nervous system through immune, neuroendocrine, and neural mechanisms. The gut microbes modulate the central nervous system (CNS) principally through neuroimmune and neuroendocrine mechanisms in which the vagus nerve and specific active metabolites are involved [4,5,6]. The gut microbiota can produce several neuroactive molecules, including serotonin, dopamine, gamma-aminobutyric acid (GABA), tryptophan (TRP), and short-chain fatty acids (SCFAs) [7]. The neuroactive compounds and hormones can influence locally the gut epithelium physiology and can go into the bloodstream to reach the brain. On the other hand, the brain can modulate the gut microbiota composition and function mainly through the autonomic nervous system and the hypothalamic–pituitary–adrenal (HPA) axis by changing the gut motility, the gut permeability, and the secretion of mucus and hormones [4,7].

In humans, the dysfunction of the GBA and the composition of the gut microbiota are closely associated with various psychiatric pathologies, including depression, anxiety, autism, and neurodegenerative and neuroinflammatory disorders [8,9,10,11]. Li et al. [12] proved the role played by the gut microbiota in the cognitive decline associated with typical aging in rats. In dogs, scientific evidence on the relationship between microbiota and neurodegenerative diseases remains limited, while they may impact the quality of life of both dogs and humans. However, recent studies have begun to investigate the connection between the gut–brain axis (GBA) and microbiota composition. Aggressive behavior in shelter dogs has been associated with distinct patterns in the gut microbiome [13,14]. Craddock et al. [15] studied a population of working dogs in which certain microbiome markers were related to performance (motivation, aggression, cowardice/hesitation, sociability, and obedience). Additionally, a review was recently published exploring the use of prebiotics and probiotics solutions linked to canine anxiety [16]. On the other hand, neurodegenerative disorders in dogs, such as canine cognitive dysfunction and canine multiple system degeneration, have been studied as homologous pathologies of Alzheimer’s disease and Parkinson’s disease in humans [10]. Interestingly, a recent review summarized the key scientific findings on the connection between behavioral disorders and the gut microbiota in dogs, including similar findings in humans and rodent models [17].

Despite growing interest in the gut–brain axis (GBA), there is a significant gap in research exploring the relationship between gut microbiota and the GBA in aged dogs. The primary aim of this study is to investigate the role of age and gut microbiota in the GBA by analyzing various biomarkers of intestinal health and behavior across different age groups of healthy Beagle dogs. Specifically, the study examines gut health through multiple blood and fecal biomarkers alongside behavioral indicators assessed through saliva, blood analysis, direct observations, and activity monitoring. These findings will contribute to the development of strategies to support gut health and overall well-being in senior dogs.

## 2. Materials and Methods

### 2.1. Animals

This study included 37 Beagle dogs grouped in junior (J), adult (A), and senior (S) categories depending on their age: up to 2 years old (*n* = 10), from 2 to 7 years old (*n* = 16), and over 7 years old (*n* = 11). Both sterilized females and males were included. The inclusion criteria consisted of Beagle dogs sterilized individuals in good health. Health was evaluated through a clinical examination performed by veterinarians and a blood analysis, which included a complete blood count and a biochemical panel. The biochemical analysis included albumin, alkaline phosphatase, total bilirubin, calcium, creatine kinase, cholesterol, creatinine, fructosamine, aspartate aminotransferase, alanine aminotransferase, globulins, phosphorus, proteins, albumin/globulin ratio, reticulocyte hemoglobin content, IDEXX SDMA™, triglycerides, and urea. The exclusion criteria comprised existing clinical diseases or any pharmacological treatments potentially interfering with the studied biomarkers based on the literature. Parasitology coprological tests were conducted on a routine basis to ensure the health status of animals regarding the presence of fecal parasites and derived clinical signs. No antibiotics, prebiotics, probiotics, postbiotics, or deworming treatments were administered during the sampling period, including a four-week washout period before starting the sampling. Dogs defecating watery, soft, or unformed stools for three consecutive days were excluded from the study.

The animals were housed in the same experimental facilities (Affinity Nutrition Center, Masquefa, Spain) under similar husbandry conditions. They lived in pairs, and they had free access to the outdoor and indoor part of their kennels (15 m^2^). They also shared a bigger outdoor space for about four hours per day where they could socialize with a stable larger group of Beagles. During this time, the facilities were cleaned and disinfected. After the outdoor activity, the dogs came back to their kennels, and they were fed once with a commercially dry, complete, and balanced diet adequate to their needs. Daily rations were calculated following the energy requirements for each dog. The feeding routine varied between dogs: some dogs rotated their diets less than every 14 days, while others had a stable diet over time. The dogs were weighed weekly to monitor the maintenance of a stable body weight. Non-special diets were offered because of the study. Considering the following diet composition range—7–8% moisture, 22–30% protein, 12–21% fat, and 1–5% crude fiber—mainly all the dogs (95%) ate diets within the appropriate range. Water supply was offered ad libitum 24 h per day.

### 2.2. Sample Collection: Feces, Blood, and Saliva

The samples collected for assessing intestinal health and behavioral markers came from feces, blood, and saliva.

Feces: Fecal samples were collected over a three-month period (March, April, and May) without disturbing the dogs’ daily routines (Table 1). The animals were not individually separated or placed in cages for sample collection. Instead, the method of direct observation was employed to identify individual fecal samples. Samples were collected while the dogs were in their kennels or when they were outdoors at their parks.

A single stool sample was collected from each dog and evaluated on a five-point scale to assess fecal consistency: 0 = watery stool, 25 = soft unformed stool, 50 = soft-formed and moist stool that retains its shape when collected, 75 = hard-formed stool that remains somewhat soft, and 100 = hard dry stool. This scale has already been applied in previous research studies [18,19]. If the fecal consistency was at or below 50, the fecal score (FS) was recorded, but the sample was not collected, awaiting another sample with a higher FS. The entire stool or a sufficient amount to fill a sterile fecal collection tube of 250 mL was collected, ensuring the exclusion of any external contaminants such as stones, grass, or sand. Within 4 h after excretion, all samples were processed using sterile, disposable materials. Following the specific requirements for the analysis, the collected fecal samples were divided into two subsamples.

The first subsample was prepared for microbiota analysis, with a minimum quantity of 0.5 g of feces introduced into a sterile 1.5 mL tube free of RNAse. The microbiota analysis was performed by Illumina sequencing of the bacterial 16S rRNA genes using primers 515F (5′ GTGYCAGCMGCCGCGGTAA) [20] and 806RB (5′ GGACTACNVGGGTWTCTAAT) [21] at the MR DNA laboratory (Shallowater, TX, USA). Sequence processing and analysis were carried out using the Quantitative Insights Into Microbial Ecology 2 (QIIME 2) v2021.8 pipeline [22]. The sequences were demultiplexed, and an ASV table was generated using DADA2 [23]. Prior to downstream analysis, sequences classified as chloroplasts, mitochondria, and low-abundance ASVs (comprising < 0.01% of the total reads) were filtered out. All samples were rarefied to even sequencing depth, based on the lowest read depth of samples, to 21,025 sequences per sample. Alpha diversity was assessed using Chao1 (richness) and Shannon diversity indices with QIIME2. Beta diversity was calculated using both unweighted and weighted UniFrac distance metrics (phylogeny-based measures) and the Bray–Curtis dissimilarity metric, with results visualized in Principal Coordinate Analysis (PCoA) plots generated in QIIME2. For the microbiota analysis, the previous scientific nomenclature for classifying different taxa was considered; the same nomenclature was used when writing the current paper. Quantitative PCR assays were used to target total bacteria, *Faecalibacterium* spp., *Turicibacter* spp., *Escherichia coli*, *Streptococcus* spp., *Blautia* spp., *Fusobacterium* spp., and *Clostridium hiranonis*. From this panel of bacterial groups, the Dysbiosis Index (DI) was calculated. This validated algorithm was used to assess a potential intestinal dysbiosis, where a DI value of <0 is categorized as normal, a DI between 0 and 2 indicates a mild to moderate shift in microbiota composition, and a DI >2 is classified as significant dysbiosis [24].

The second subsample was prepared for the analysis of SCFA concentration and proportion, canine calprotectin (cCP), and immunoglobulin A (IgA) concentrations. In this case, 5 g of feces were pooled in a sterile stool tube. Both types of tubes were carefully filled and stored at −80 °C until they were dispatched for analysis at the Small Animal Clinical Sciences Department located at Texas A&M University, College Station, TX, USA.

Saliva and blood: Saliva and blood samples were collected from approximately 8:30 to 12:30 am. All the dogs were previously fasted for 8 h to avoid any interference in the measurements. Initially, the dogs were moved to a familiar area close to their kennels. Within the first five minutes, saliva samples were collected by rubbing a synthetic swab specifically designed for diagnostic saliva collection (Salivette cortisol tubes; Sarstedt, Germany) on the dogs’ tongue and the inside surface of their cheeks. The swab was then promptly returned to the saliva collection tube. Subsequently, blood sampling was conducted through jugular vein venipuncture. The total volume required was 5.5 mL:0.5 mL of complete blood (EDTA blood collection tube) for a complete cell blood count (CBC) analysis and 5 mL collected for analyzing various serum biomarkers.

After the sampling, the saliva and blood samples were prepared as follows for analysis. The saliva collection tubes were centrifuged at room temperature at 1420 G for 10 min. The swab was removed, and the saliva was extracted and transferred from the tube to a 1.5 mL tube (Eppendorf). The minimum volume needed for salivary cortisol (CORT) and oxytocin (OT) analysis was 300 µL of saliva. The Eppendorf tubes were then stored at −80 °C until shipment to the lab (Interdisciplinary Laboratory of Clinical Analysis, Murcia, Spain). OT concentration was determined using the validated method for dogs AlphaLISA monoclonal assay [25]. CORT concentration was analyzed using the Siemens Immulite automated chemiluminescence assay.

Serum collection tubes were set at room temperature for 30 min and centrifuged at 1420 G for 10 min. The serum volume obtained after centrifugation was approximately 1.3 mL, divided into four subsamples: two 500 µL subsamples, one 200 µL subsample, and one 100 µL subsample. Three subsamples were stored at −80 °C. One 500 µL subsample was sent to be refrigerated, along with the EDTA blood collection tube, to Idexx laboratory (Barcelona, Spain) for a biochemistry profile and to assess thyroxine levels (TT4). The 200 µL subsample was used to analyze cobalamin (B12) and folate (B9) concentrations by using chemiluminescence assays (Immulite 2000 Vitamin B12; Folic Acid, Siemens Medical Solutions Diagnostics, Erlangen, Germany) in the Small Animal Clinical Sciences Department located in Texas A&M University (College Station, TX, USA). In the 100 µL subsample, tryptophan and kynurenine (KYN) concentrations were determined using an L-Tryptophan ELISA kit (ImmuSmol, ref. BA E-2700, Bordeaux, France) and an L-Kynurenine ELISA kit (ImmuSmol, ref. BA E-2200, Bordeaux, France). An EMS Reader MF V.2.9-0 was used to read the ELISA plates (Clinical biochemistry laboratory at the Autonomous University of Barcelona, Barcelona, Spain). The last 500 µL subsample stored at −80 °C was sent to determine canine C-reactive protein (CRP) and haptoglobin (HP) concentrations by immunoturbidimetric assays validated for dogs [26,27] (Interdisciplinary Laboratory of Clinical Analysis, Murcia, Spain).

### 2.3. Behavioral Observations and Activity

Behavioral observations were conducted one week after the saliva and blood sampling to avoid interferences. The observations were performed by the same trained observer, and all behaviors were classified according to a predetermined ethogram (Table 2). The behavior was measured both in their kennels and in their outdoor parks, where dogs spent around 20 h and 4 h, respectively. Regarding the observations in the kennels, the animals were recorded during the morning and the afternoon. One hour was selected for each period to avoid interference with daily routines, such as feeding, outdoor time, or cleaning. Each 60 min period was divided into intervals of 10 min and scan observations were conducted for each interval. In total, twelve scans were performed per animal, and twelve behaviors were assigned to each animal. The area recorded was only the indoor one; if the animal was in the outdoor area of the kennel, the observation was considered null. The behavioral assessments in the outdoor parks were performed for 60 min and repeated on 2 different days. In total, twelve scans were performed per animal assigning twelve different behaviors to each animal. A null observation was recorded when any park structure obstructed a clear view of the animal, making it difficult to properly categorize its behavior.

For the activity measurements, a physical activity actigraphy monitor (Actical^®^, Philips Respironics, Bend, OR, USA) was placed on the dogs’ collars for 4 days to measure their activity during three consecutive days. The first day was not recorded since this period was left for adaptation. Dogs were previously trained to wear the device. The activity was retrieved as total activity (TAC) (24 h) and then divided into diurnal activity (DAC) (from 7:00:00 to 20:59:59) and nocturnal activity (NAC) (from 21:00:00 to 6:59:59).

### 2.4. Definition of the Study Variables

The primary explanatory variable was defined as the age category, either junior, adult, or senior. The model was adjusted using several demographic variables, including body weight, sex, feeding routine, and housing. The feeding routine consisted of two categories: “rotation”, indicating animals that changed their diet type within periods shorter than two weeks, and “stable”, indicating animals that consistently consumed the same diet for at least four weeks. Each animal was exclusively assigned to one category throughout the entire study. Housing was the variable used to identify the two different buildings where animals were housed.

The response variables were classified into three groups: biomarkers of intestinal health, behavioral observations, and activity measurements. The intestinal health biomarkers measured in feces included microbiota composition using the 16S rRNA method, qPCR results for absolute quantity of total bacteria, *Faecalibacterium*, *Turicibacter*, *Streptococcus*, *E. coli*, *Blautia*, *Fusobacterium*, *Clostridium hiranonis*, *Bifidobacterium*, *Bacteroides*, *Lactobacillus*, and the Dysbiosis Index (DI) [27]. Within the same category of intestinal health biomarkers, alpha diversity (Chao 1 and Shannon diversity), FS, SCFA concentrations in fecal dry matter (μmol/g), SCFA relative amounts (%), cCP (ng/g), and IgA (mg/g) concentrations were also analyzed. The intestinal health biomarkers measured in blood serum included B9 (ng/mL), B12 (pg/mL), CRP (μg/mL), HP (g/L), TT4 (μg/dL), TRP (μg/mL), and KYN (ng/mL) concentrations.

For the second group of response variables, defined as behavioral indicators, salivary CORT (μg/dL), salivary OT (pg/mL) concentrations, and behavioral observations were included. Regarding the observations, the relative frequencies of the different defined behaviors were calculated for statistical analysis. Finally, activity measurements completed the set of response variables considered for statistical analysis.

### 2.5. Statistical Analysis

For the general statistical analysis, thirty-five dogs were considered, as the behavioral markers were missing in two senior dogs. For the descriptive statistics, quantitative variables were assessed using mean and standard deviation, while qualitative variables were examined through relative and absolute frequencies. Pearson’s (or Spearman’s) correlations were assessed between age, considered as a quantitative continuous variable, and the other parameters.

Then, differences among age categories were studied by applying the appropriate test according to the distribution of the variable (ANOVA; Kruskal–Wallis test), with the null hypothesis assuming equality between groups. The compliance of application criteria was verified using Shapiro–Wilk’s normality test.

To investigate the relationship between age categories and the studied variables, the appropriate linear model was performed, including body weight, sex, feeding routines, and housing as potential explanatory variables. The estimated marginal means (emmeans) and standard error (SE) for the age categories were calculated for each variable using the adjusted model. Finally, the post hoc comparisons were performed through the Pairwise analysis. The relation between the response variables and the age as a quantitative variable was analyzed using Spearman’s correlation.

The correlation between gut health biomarkers and behavioral indicators (biomarkers of behavior, behavioral observations, and activity) was evaluated using pairwise Spearman correlations. These correlations were studied considering all the animals as a group (including the three age categories) and then split by age category. This statistical approach allowed for the evaluation of potential interactions between age and the markers of interest and, more precisely, to detect different age patterns when studying the possible association between gut health biomarkers and behavioral indicators. The microbial groups included in the correlations were based on the qPCR analysis.

For the microbiota statistical analysis (16S rRNA sequencing analysis), the ANOSIM (Analysis of Similarity) test within the PRIMER 7 software package (PRIMER-E Ltd., Luton, UK) was employed to investigate the differences in microbial communities and the size effect (R-values ranging from 0 to 1, with a higher R-value indicating a larger size effect) between the defined age categories. The normality of all datasets was assessed using the Shapiro–Wilk test (JMP Pro 11, SAS Software Inc., Cary, NC, USA). Subsequently, the Kruskal–Wallis test was conducted (Prism v.9, GraphPad Software Inc., La Jolla, CA, USA), followed by post hoc analysis using Dunn’s multiple comparison test to identify age category differences in bacterial taxa. To control for multiple comparisons, all *p*-values were adjusted using Benjamini and Hochberg’s False Discovery Rate [28] at each taxonomic level. In the statistical analyses, significance was established at a *p*-value < 0.05, while a *p*-value < 0.1 was considered a trend when the post hoc analysis was significant (*p*-value < 0.05). The *p*-values derived from the 16S rRNA sequencing analysis underwent adjustment using a false discovery rate (FDR) of 0.05 (q-value).

## 3. Results

### 3.1. Description of the Dog Cohort

The thirty-seven dogs in the cohort were distributed similarly between the junior and senior categories, although the number of animals in these categories was lower than in the adult category (junior: J, *n* = 10, adult: A, *n* = 16, and senior: S, *n* = 11). The mean body weight was slightly higher in seniors (13.4 ± 1.6 kg) compared to juniors (12.3 ± 1.5 kg) and adults (12.8 ± 1.9 kg). Across the cohort, most dogs were neutered females (72.97%), with neutered males comprising 27.03%; notably, all senior dogs were female. Regarding feeding routines, the majority followed a stable routine (64.86%), although rotational feeding was more prevalent in adults (50%) than in juniors (10%) or seniors (36.4%). Housing distribution showed more dogs living in building P (62.16%) compared to building D (37.84%), a pattern consistent across age categories (Table 3).

### 3.2. Biomarkers of Intestinal Health

#### 3.2.1. Quantitative Real-Time PCR and 16S rRNA Sequencing Analysis

The qPCR results analysis are shown in Table 4. *Bacteroides’* relative abundance was higher in S than in A and J; these results are also supported by the 16S analysis. *Turicibacter* was significantly negatively correlated with age (rho = −0.365; *p*-value < 0.05), but the results were not significant in the multivariate analysis. *Faecalibacterium* and *Blautia* log DNA copies tended to differ (*p*-value < 0.1) according to the age category. Those results were confirmed by 16S rRNA sequencing, as relative abundances of those two bacteria significantly differed according to the age category, with *Faecalibacterium* relative abundance being higher in S compared to A (*p*-value = 0.002; q-value = 0.010) while *Blautia* relative abundance higher in J compared to A (*p*-value = 0.043).

The dysbiosis index and the alpha diversity indices (Chao1 and Shannon) were not significantly different between age categories. The dysbiosis index values were within the normal range; only two adult dogs were within the dysbiosis index range, defined as mild to moderate microbiota shift (DI between 0 to 2).

**Figure 1:**
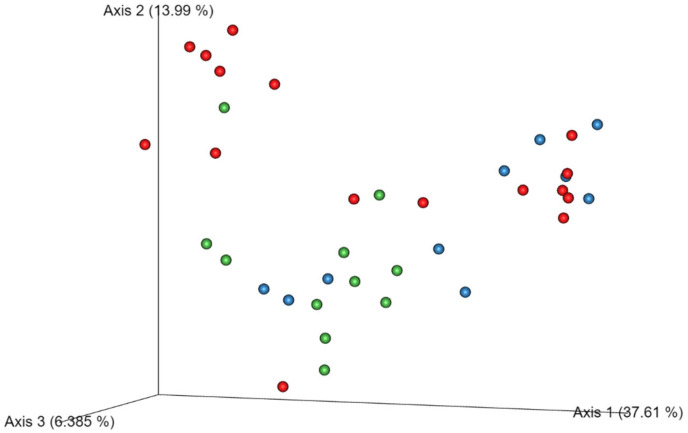
Three-dimensional representation of unweighted Unifrac distance of the microbiota communities in the studied dog population. Different age groups are represented with different colors: green (senior), red (adult), and blue (junior).

Microbial communities were significantly different based on Unweighted UniFrac analysis, which solely assesses the presence or absence of individual taxa, were significantly different between S and J categories (Figure 1) but were not significant based on the weighted Unifrac and Bray–Curtis analysis, corresponding to the results of the DI (Table 5).

At the phylum taxonomic level, Firmicutes, Actinobacteria, Fusobacteria, Bacteroidetes, and Proteobacteria were identified (Table 6). Firmicutes showed the highest relative abundance, while Proteobacteria exhibited the lowest. Significantly different relative abundances were observed for Actinobacteria, Bacteroidetes, and Proteobacteria between age groups. Specifically, Bacteroidetes and Proteobacteria phyla were relatively more abundant in the oldest group, while Actinobacteria showed a lower percentage in S.

At the family level, the relative abundance of *Bifidobacteriaceae* (Actinobacteria) was higher in A compared to J. *Bacteroidaceae*, and *Prevotellaceae* families (Bacteroidetes) were relatively more abundant in the S category than in A and J. [Paraprevotellaceae] relative abundance was higher in S compared to A. Within Firmicutes, *Lachnospiraceae*, *Peptococcaceae*, Uncl. *Clostridiales I*, *Ruminococcaceae*, and *Veillonellaceae* families exhibited significantly different relative abundances between age categories. *Succinivibrionaceae* (Proteobacteria) were relatively more abundant in S than in A, and *Alcaligenaceae* was relatively higher in S compared to J. See the detailed information in the Supplementary Material (Table S1).

#### 3.2.2. Fecal Score, SCFAs, IgA, and cCP

FS values were not significantly different between the age categories and were all within the normal range (67.46 ± 4.26; 67.82 ± 3.00; 72.15 ± 5.11; for J, A, and S, respectively).

For the SCFA concentrations, acetate was the most abundant one in the feces and it was significantly higher in S and A dogs compared to J (Table 7). Both acetate and total SCFA concentration showed significant positive correlations with age (rho = 0.358 and 0.365; *p*-value = 0.05). Total SCFA content was different, only showing a trend (*p*-value = 0.055) between groups, but was significantly different when comparing A vs. J (*p*-value = 0.017). Butyrate concentration was higher in A (*p*-value = 0.071), being significantly different in the post hoc analysis compared with J (*p*-value = 0.036).

Considering the relative proportion of SCFAs, propionate showed a significantly lower percentage in S and A compared with J (3.35%, 3.65%, and 4.94%, respectively). For the cCP and IgA analysis, no significant difference was found.

#### 3.2.3. Serum Haptoglobin, C-Reactive Protein, Folate and Cobalamin

B9 concentration (ng/mL) was higher in S compared to A and J categories. HP concentration correlated positively with age (rho = 0.426; *p*-value = 0.05). On the other hand, B12 concentration was negatively correlated with age (rho = −0.424; *p*-value = 0.05). However, B12, CRP, and HP concentrations did not show any difference in the multivariate analysis when comparing the age categories (Table 8).

### 3.3. Biomarkers of Behavior

For the results of the behavioral indicators based on blood and saliva, no differences were found between the age categories, except for TT4, which exhibited lower levels in the A and S groups compared to the J group (Table 9).

### 3.4. Behavioral Observations and Activity

In the analysis of behavioral observations, distinct patterns were identified among the age categories (Table 10). During park observations, P behavior exhibited significantly higher levels in category J compared to both categories A and S (*p*-value < 0.001 and *p*-value = 0.001, respectively). HA behavior was more prevalent in category A than in category J (*p*-value = 0.005). Furthermore, when observations were conducted in the kennels, category J displayed significantly higher levels of exploratory behavior compared to both categories A and S (*p*-value = 0.048 and *p*-value = 0.013, respectively). Regarding the activity measurements, TAC showed a negative correlation with age (rho = −0.474; *p*-value < 0.05). Similarly, DAC demonstrated an inverse correlation with age (rho = −0.502; *p*-value < 0.05). But no differences between age categories were described in the multivariate analysis.

### 3.5. Correlations Between Intestinal Health Biomarkers and Behavioral Indicators

The relationship between gut health biomarkers and behavioral indicators (biomarkers of behavior, behavioral observations, and activity) and the detection of different age patterns is detailed in Supplementary Material (Table S2). Considering the biomarkers of behavior: *Turicibacter* negatively correlated with TRP considering all the dogs, and this negative correlation was specifically marked in A. *Blautia* and *Clostridium hiranonis* correlated positively with thyroxine. *Lactobacillus* correlated positively with saliva CORT concentration, while fecal acetate, propionate, butyrate, and total SCFA concentrations were negatively correlated with blood TT4 concentration. Isobutyric and isovaleric acid concentrations were negatively correlated with blood KYN and saliva CORT concentrations. Blood KYN followed the same pattern when paired with fecal cCP. And blood HP positively correlated with blood TRP concentrations, the same direction was followed within the different age categories, being significant only for A. With regard to the behavioral observations and activity, only the significant correlations influenced by age categories are discussed: the fecal DI correlated positively with DAC, following significantly the same direction in the A category. Bacteroidetes correlated negatively with TAC and DAC showing an age association in the S and A category, respectively. On the contrary, *Streptococcus* and *Blautia* positively correlated with TAC and DAC, which was also reflected when the A category was studied. *Bifidobacterium* relative abundance positively correlated with resting behavior, with a high correlation with age in the J category. FS correlated positively with grooming behavior, being significantly correlated within the J category. 

## 4. Discussion

The number of publications in the field of fecal microbiota in dogs is significantly increasing. Previously published works in canine science examine the relationship between intestinal microbiota and various pathologies, especially those related to the gastrointestinal system [29,30,31,32,33]. Additionally, there is a growing number of publications studying the gut microbiome related to specific ’biotic’ supplements [34,35,36,37,38,39,40,41] and different diets composition [42,43,44]. However, the number of studies that exploratorily investigate intestinal microbiota and biomarkers of intestinal health, as well as their relationship with other organs and systems (microbiota–gut–organ axis concept), remains limited. This publication aims to generate knowledge about the gut–brain connection in dogs, including biomarkers of intestinal health (to assess the gut part of the axis) and biomarkers of behavior (to evaluate the brain part of the axis). In addition, the study includes a cohort of healthy animals of different ages in which differences due to aging are explored.

### 4.1. Biomarkers of Intestinal Health

#### 4.1.1. Quantitative Real-Time PCR and 16S rRNA Sequencing Analysis

The DI showed no significant differences among the age categories. Although different age dogs were included in the study, no differences were expected between healthy individuals. This was confirmed by weighted Unifrac and Bray–Curtis analysis in the 16S rRNA gene sequencing data. Similarly, alpha diversity, which measures diversity within a specific sample, did not exhibit significant variations. This suggests that the overall microbial diversity within each group was comparable, as previously described [19]. The significant differences observed in Unweighted UniFrac between seniors and juniors imply differences in the presence of individual bacteria taxa, which may indicate potential age-related small variations in the composition of community structures.

In terms of microbiota relative abundance, Firmicutes, Actinobacteria, Fusobacteria, Bacteroidetes, and Proteobacteria were the five phyla identified. Previous research supports that the majority of bacterial sequences found in the canine gastrointestinal tract belong to these five distinct phyla [45,46,47]. In our study, the three main bacterial groups reported were Firmicutes, Actinobacteria, and Fusobacteria, and this distribution was observed across the three age categories. Previous studies found a different distribution in the three main phyla, with Bacteroidetes appearing in place of Actinobacteria [48,49]. Considering the characteristics of our studied population, we hypothesize that the observed differences may result from variations in the analysis methodology or specific traits of the study cohort, which share some common conditions (biotypes).

In our study, Bacteroidetes and Proteobacteria were relatively more abundant in the oldest group, whereas Actinobacteria were less abundant. Bacteroidetes are involved in the digestion of complex carbohydrates and play a role in maintaining gut health. Lower relative abundances of this phylum have been described in dogs with inflammatory bowel disease (IBD) [31]. Additionally, previous studies in senior dogs already described differences in lower relative abundance of *Bacteroides* compared to younger dogs [50,51]. In our study, this phylum exhibited a higher relative abundance in the older population, mainly caused by the results observed in the families *Bacteroidaceae*, *Prevotellaceae*, and *[Paraprevotellaceae]*, as well as in the genus *Bacteroides* (qPCR and 16S analysis). These findings suggest that small changes may occur in the Bacteroidetes phylum with age; however, these changes may not always follow the same direction, especially when no gastrointestinal pathologies are linked to the aged study population. Based on our findings, Proteobacteria was more abundant in the oldest category. This increase was associated with a higher relative abundance of both the *Succinivibrionaceae* and *Alcaligenaceae* families. These results are consistent with our previously published data from a larger cohort of dogs [19]. Given the association in the literature between an increase in Proteobacteria and gut inflammation [52], we hypothesize an association between the higher levels of Proteobacteria observed in older dogs and the process of inflammaging, a chronic and progressively proinflammatory state that develops in mammals as they age [53].

#### 4.1.2. Haptoglobin, C-Reactive Protein, Folate, and Cobalamin

Serum HP, CRP, B9, and B12 serve as indicators of various physiological processes and health conditions, including inflammation disorders, infections, hepatic and cardiovascular diseases, as well as the synthesis of neuroactive compounds [54,55,56,57,58,59]. In our study, folate levels were higher in the senior category and correlated positively with age. Almost all the study dogs harbored concentrations within the normal range described by the laboratory (7.7–24.4 µg/L), and only two senior dogs had levels slightly above this range. Hyperfolatemia has been linked to intestinal inflammation in dogs, including specific conditions such as chronic enteritis [60]. Consequently, we hypothesized that the elevated levels of folate observed in older dogs could be linked to the inflammatory process associated with aging [53]. Interestingly, in our study, we found a positive correlation between folate and haptoglobin levels. In the case of haptoglobin, all values were within the normal range [23]. Although haptoglobin levels correlated with age, no differences were described between the age categories. In addition, no significant finding was described linked to the C-reactive protein analysis. Although we did not find any differences and there is limited knowledge about acute-phase proteins and aging in dogs, it is noteworthy that various acute-phase proteins, including C-reactive protein, have been reported to be higher in elderly humans compared to younger individuals [61].

In our population, the cobalamin levels were within the normal range in all groups [30,60]. The values showed a negative correlation with age, suggesting that older dogs may have reduced efficiency in their absorption. Cobalamin absorption is a complex process occurring along the gastrointestinal tract. A pathological condition in this organ may be causing hypocobalaminemia, a condition that also appears to be more prevalent in older age groups [30]. In our study, mild intestinal inflammation possibly related to aging could explain the negative correlation between cobalamin and age. Interestingly, both concomitant conditions, hypocobalaminemia and hyperfolatemia, could also be explained by bacterial consumption of cobalamin and the overproduction of folate synthesized by bacteria [62,63]. This relationship has been previously described in dogs with small intestinal dysbiosis [60]. Additionally, the measurement of methylmalonic acid could help to better understand this relation. Methylmalonic acid is implicated in key processes of metabolic reprogramming and cellular signaling pathways [64]. It is a more sensitive marker than cobalamin and has already been associated with pathologies linked to the aging process [64,65,66].

#### 4.1.3. Fecal Consistency, SCFAs, Immunoglobulin A, and Calprotectin

The fecal consistency of the animals included in the study fell within the normal range, and there were no age-related differences. These results were as expected since the study was conducted in a cohort of healthy animals where gastrointestinal signs were not present.

The analysis of SCFAs was conducted mainly to assess the potential differences in the fermentation activity. In our study, acetate was the most abundant SCFA; it was significantly different between the age categories and positively correlated with age. For the total SCFA analysis, a positive correlation with age was described. The SCFA results showed a higher fermentation activity in the senior group, which was consistent with the microbiota composition, revealing an increase in the relative abundance of bacteria able to use complex carbohydrates. This higher activity may be explained by diet composition and type, a lower upper tract digestibility of macronutrients, the presence of specific pathologies, the microbiota composition, and also by age. In our study, two dogs belonging to the senior category were fed diets out of the composition defined range (7–8% moisture, 22–30% protein, 12–21% fat, and 1–5% fiber). One senior dog was fed a high-fiber diet (8.0% moisture, 23% protein, 12% fat, and 6.5% fiber), while the other one was fed a low-protein diet (7.5% moisture, 14.5% protein, 17.5% fat and 3% fiber). All dogs were eating dry food diets. The presence of pathology or dysbiosis was not previously diagnosed in any of the dogs enrolled in this study. Finally, microbiota composition and age were identified as the major potential explanatory factors for fermentation activity in this study. For example, increased colonic butyrate concentrations have been described in senior dogs [67], as also observed in our study. Moreover, in our study, *Faecalibacterium’s* relative abundance tended to be higher in seniors, probably contributing to butyrate production due to its well-known butyrogenic capacity. In contrast, propionate levels as a percentage (relative amount) were found to be lower in senior and adult dogs compared to juniors. This condition may be explained by the relative proportion calculation. Since acetate plays an important role in this calculation, its higher concentration in seniors and the lower concentration in juniors may explain the overrepresentation of propionate when expressed as a relative amount.

Fecal concentrations of canine calprotectin and immunoglobulin A are described in the literature as non-invasive biomarkers for assessing gut health in dogs. Fecal cCP has been linked to intestinal inflammation, while IgA is associated with gut-immune homeostasis [68,69]. In our study, IgA and cCP levels showed no significant changes across different age categories. Interestingly, both biomarkers have been previously correlated with age in rodents, dogs, and humans, although the results concerning cCP remain inconsistent [70,71,72]. Further research is needed to determine if these two fecal biomarkers can be considered reliable indicators of aging in dogs.

### 4.2. Biomarkers of Behavior

Tryptophan is an essential amino acid involved in various metabolic processes, including the synthesis of neuroactive molecules such as melatonin and serotonin. kynurenine, a metabolite of tryptophan, is produced via the kynurenine pathway, which represents a major route for tryptophan metabolism [55]. Alterations in the levels of tryptophan, kynurenine and in the kynurenine/tryptophan ratio may indicate disruptions in tryptophan metabolism due to various factors, including age [73,74]. In our study, while tryptophan levels were numerically similar between the groups, both kynurenine levels and kynurenine/tryptophan ratio increased with age. A numerical effect was observed for the kynurenine, but the intra-individual variability and the number of dogs were probably too low to detect a statistical difference. However, any of these biomarkers showed significant differences across the age groups. Thus, the relationship between age, tryptophan, kynurenine, and their ratio observed in this study differed from our expectations based on previous research [73].

Cortisol and thyroxine are bioactive compounds that participate in the HPA axis. This axis is crucial in mediating the stress response and regulating the interaction between the gut and the brain [75]. According to Taszkun et al. [76], thyroid hormone levels in healthy dogs can vary based on factors such as age, sex, breed, and physical activity. Consistent with this, our study found that TT4 levels were higher in the younger age group. Although higher cortisol levels have been associated with aging [77], our study did not observe any significant age-related changes in the dogs’ cortisol levels.

Oxytocin is a neuropeptide primarily synthesized in the hypothalamus, playing a key role in various physiological and emotional processes and being linked to the gut–brain axis [78,79]. Recent research has associated oxytocin with cellular aging across several systems, including muscle, bone, skin regeneration, and hippocampal function [80,81,82,83,84]. In our study, we hypothesized that this biomarker would vary, particularly in the older group. However, no age-related differences were observed. We speculate that significant changes in oxytocin levels might only become apparent in dogs at the very end of their lives, possibly showing clinical signs of advanced aging. Nevertheless, the role of oxytocin in aging remains unclear and warrants further investigation, especially for its potential in anti-aging therapies.

### 4.3. Behavioral Observations and Activity

The gut microbiota can influence behavior and activity levels through various pathways, including neurotransmitter and metabolic production, neuroendocrine and immune modulation, and vagus nerve stimulation [3]. Assessing changes in behavior and activity may provide insights into the relationship between the gut and the brain [9,75]. Our behavioral observations suggest several age-related variations, with younger individuals generally being more active, as previously described in the literature [85,86]. Specifically, younger individuals appeared more engaged in playing when at the park, likely taking profit from the presence of a larger group of conspecifics, and were more exploratory when being in the kennel with their mates. Conversely, high-alert behavior observed in the park was less pronounced in the junior group, which may be related to their engagement in playing.

### 4.4. Correlations Between Intestinal Health Biomarkers and Behavioral Indicators

The negative correlation between tryptophan and *Turicibacter* suggests a potential role for this bacterial genus in tryptophan metabolism. *Turicibacter* may be involved in the breakdown or utilization of tryptophan, potentially influencing the availability of this essential amino acid for serotonin synthesis. Various bacterial groups have been identified as key contributors to the bacterial fermentation of tryptophan in the gastrointestinal tract, producing indoles such as indole-3-acetic acid, indole-3-propionic acid, indole-3-lactic acid, indole-3-aldehyde among others [87,88,89]. Specifically, *Turicibacter* seemed to be involved in both the kynurenine and the indole pathways [90]. However, interestingly, no correlation was found between *Turicibacter* and the kynurenine levels. There is intense competition between serotonin, indole, and kynurenine for the available tryptophan, and this increases the complexity of understanding the tryptophan-related metabolites [90]. Additionally, *Turicibacter* has also been associated with anti-inflammatory properties, influencing host bile acid and lipid metabolism and promoting the intestinal production of 5-HT [91]. All of this could have implications for behavior and cognitive function, making it particularly interesting for senior dogs due to its potential role in cognitive impairment. However, further research is needed, as the specific mechanisms underlying these relationships and their potential impact on serotonin levels are not well described in the literature.

The positive correlation between thyroxine and *Blautia* and *Clostridium hiranonis* is described for all the animals. Interestingly, within the senior category, *Clostridium hiranonis* showed a significant correlation with thyroxine levels. *Clostridium hiranonis* has an important role in maintaining a healthy microbiota in dogs [24,92]. It has been identified as a key player in the conversion of primary to secondary biliary acids, being associated with lipid metabolism and the endocrine system [46,93,94]. With all of this, we could consider a potential association between this bacterial group and thyroid hormone levels. In our study, we observed a negative correlation between acetate, propionate, butyrate, and total SCFAs with thyroxine. The acetate correlation with thyroxine was especially important in adults and seniors, while in the case of propionate was in seniors. Although the impact of SCFAs on host thyroid function has already been described and reviewed [95], the specific mechanisms are still unclear. SCFAs have been shown to modulate thyroid hormone production and signaling, potentially through effects on the hypothalamus-pituitary-thyroid axis. This finding suggests that the gut microbiome, through SCFA production following different patterns at different life stages, may play a role in regulating thyroid hormone levels and potentially influencing behavior. In addition, the positive correlation between salivary cortisol and *Lactobacillus* suggests a potential link between this bacterial genus but unrelated to a specific life stage. *Lactobacillus* is often associated with beneficial effects on gut health and stress modulation. Specific species and strains of *Lactobacillus* have shown a positive effect on modulating salivary cortisol levels and other stress-related indicators in rats and humans [96,97]. More generally, the most extensively studied group linked to the gut–brain axis is *Lactobacillus*, mainly due to its therapeutic potential. Lactobacilli are also well known for their probiotic properties; they modulate immune responses and support a balanced microbiota [98,99]. Nevertheless, less is known about this bacteria’s role in dogs, although several studies already mentioned positive effects. Of note, all four genera in which we identified different correlations with behavioral parameters belong to the Firmicutes phylum. Previous studies have already highlighted the significant roles this phylum plays in gut health, immunity, and behavior [24,87,88,89,91,96,97]. Those data suggest that, like in other animal species, Firmicutes and, more specifically, *Lactobacillus* may play functional roles in the GBA. Identifying key microbial metabolites related to key behavior is probably the next step to create putative causation between this bacterial group and the GBA. On the other hand, we found a negative correlation between cortisol and isobutyric and isovaleric acid, being especially noteworthy for the adult category. The relations described for cortisol, kynurenine, and isobutyric and isovaleric acids may suggest the existence of pathways between the microbial metabolites and the stress response in dogs. On the other hand, kynurenine correlated negatively with isobutyric and isovaleric acid, and a negative correlation was described for the senior dogs in the case of isovaleric acid. Overall, this suggests that microbial proteolytic activity-resulting metabolites may play a role in gut–brain communication. Interestingly, in humans, isovaleric acid and cortisol had already been correlated, as well as depression and isovaleric acid [100]. The association between depressive episodes and the dysregulation of the HPA axis has been already well established [101,102].

We found a negative correlation between kynurenine and calprotectin only when we analyzed all the animals as a group, but no association was described when the two parameters were studied by age category. This correlation could be explained in the direction of having the tryptophan metabolism altered because of an inflammatory condition in which calprotectin increases while tryptophan decreases. Serum tryptophan levels were previously described as significantly lower when comparing individuals diagnosed with a specific intestinal inflammatory condition (intestinal bowel disease) with a control group [103]. Kynurenine and the kynurenine/tryptophan ratio were also altered in individuals under intestinal inflammatory conditions [104]; however, in our study, no correlation was found. On the other hand, the positive correlation found between tryptophan and haptoglobin, particularly in adults, may suggest a potential link between inflammation, tryptophan metabolism, and behavior in adult dogs. Although there was not a correlation between the kynurenine and the seric haptoglobin, the changes in haptoglobin levels could still influence tryptophan metabolism and potentially impact serotonin production, leading to behavioral changes.

Finally, it is important to outline the study’s limitations, such as the number of animals that participated in the study, the fact that we conducted only a single sampling without tracking age progression, and the categorization by three age groups, which may not allow detection of changes at specific ages. Additionally, there are factors that could be influencing the results, such as the animals being fed different diets, even though most diets had a similar composition range. It would also have been beneficial to collect body composition values for the animals, in addition to their other demographic characteristics included in the study. We also acknowledge that the spayed/neutered status of the studied population introduces a potential bias by removing the influence of sexual hormones, which are indeed an important factor associated with age. This is an important point, particularly in relation to the extrapolation of our findings to the general dog population or broader canine aging studies. Acknowledging that correlations do not always imply causation, we believe that this study provides a foundation for further exploring the relationships between behavioral parameters and intestinal health, contributing to a more comprehensive understanding of the underlying causes and mechanisms.

## 5. Conclusions

The overall results of the studied biomarkers of intestinal health, biomarkers of behavior, and behavioral observations suggest minor changes in the different dog age groups. The observed differences in correlations between various biomarkers of gut health and behavior, particularly within the age categories, highlight the importance of considering age-related factors when studying gut health and behavioral biomarkers. However, further research is needed to better understand the mechanisms and the specific pathways involved in the relationship between the specific studied biomarkers. The findings described in this study could have implications for the development of nutritional interventions adapted to the needs of different age groups in dogs.

## Figures and Tables

**Table 1:** Detailed information about the number of fecal samples collected and their distribution over time by month. The samples are categorized by age group.

	Junior	Adult	Senior	Total
March	5	8	4	17
April	0	8	7	15
May	5	0	0	5
Total	10	16	11	37

**Table 2:** Ethogram containing the behaviors identified in the behavioral observations conducted both in the kennels and in the outdoor parks. All the behaviors described have been assessed in the kennels and in the outdoor parks.

Behavior	Abbreviation	Description
Drinking	D	The dog laps or takes up water with its tongue from the drinking bowl.
Eliminating	EL	Urinating and defecating behaviors are included in this category.
Sleeping	SL	The dog lies down, its breathing frequency decreases, and its eyes are often closed or partially closed.
Resting	R	The dog lies down but is awake. The corporal position is relaxed and not alerted.
Playing	P	Engaging in activities for entertainment involving physical movements and interactions with other inanimate objects (e.g., toys).
Exploring	EX	Investigating and interacting with the environment to gather information and satisfy curiosity. Sniffing is often present during the exploratory behavior.
Affiliative behavior	AF	Displaying actions that strengthen social bonds and promote positive interactions with conspecifics. It should be an interaction with a conspecific.
Grooming	G	Cleaning and maintaining the coat and the skin is often conducted to self-regulate (performed in a relaxed manner) or to increase social bonds between the group.
Overgrooming	OG	Excessive or compulsive self-grooming, the dog is really focused on the performance of this behavior, and the posture is tense.
Agonistic behavior	AG	Displaying behaviors associated with conflict, competition, or aggression, including growling and persecution.
Barking Defensive	BD	Vocalization is conducted together with a defensive posture that may include a lowered body, ears back, tail tucked, and a still demeanor.
Barking Offensive	BO	Vocalization is accompanied by an offensive posture that may involve a forward-leaning body position, forward-pointing ears, stiff legs, and a raised and rigid tail.
Hiding	H	Withdrawing or seeking seclusion to avoid social interactions or perceived threats. Fear and alertness behaviors are often shown.
Destructive Behavior	DB	Actions or behaviors such as chewing, digging, scratching, or other activities that result in damage to objects and structures.
Repetitive Behavior	RB	Engaging in actions or movements in a repetitive and stereotypical manner, such as pacing or circling.
High Alert	HA	Being in a state of heightened awareness and attention, often accompanied by increased vigilance and sensitivity to stimuli. The dog can be either standing or lying down.
Coprophagia	C	Consumption of dog feces.
Walking	W	Moving from one place to another with no apparent objective (e.g., chasing a dog, playing with other dogs).

**Table 3:** Description of the dog cohort, including its demographic variables such as age, body weight, sex, feeding routine, and housing, categorized by age group. Means and standard deviation (mean ± SD) are presented for quantitative variables, and absolute numbers and relative frequencies are shown for qualitative variables.

Categories	All	Junior (J)	Adult (A)	Senior (S)
Variables	*N* = 37	*n* = 10	*n* = 16	*n* = 11
Age (y.o.)	5.5 ± 4.1	1.2 ± 0.3 ^a^	4.4 ± 0.5 ^b^	11.2 ± 2.3 ^c^
Body weight (kg)	12.9 ± 1.7	12.3 ± 1.5	12.8 ± 1.9	13.4 ± 1.6
Sex				
Female (neutered)	27 (72.97%)	6 (16.22%)	10 (27.03%)	11 (29.73%)
Male (neutered)	10 (27.03%)	4 (10.81%)	6 (16.22%)	0 (0%)
Feeding routine				
Rotation	13 (35.14%)	1 (2.70%)	8 (21.62%)	4 (10.81%)
Stable	24 (64.86%)	9 (24.32%)	8 (21.62%)	7 (18.92%)
Housing				
Building D	14 (37.84%)	3 (8.11%)	7 (18.92%)	4 (10.81%)
Building P	23 (62.16%)	7 (18.92%)	9 (24.32%)	7 (18.92%)

^a,b,c^: Differences between age categories are indicated using superscript letters.

**Table 4:** Results of the quantitative real-time PCR (abundance expressed as LogDNA) and values of the calculated dysbiosis index (ratio) comparing the age categories. Within each category, values are expressed as estimated marginal means (emmeans) and standard error (SE).

	Junior		Adult		Senior		
Indicators	Emmeans	SE	Emmeans	SE	Emmeans	SE	*p*-Value
Universal Log DNA	11.04	0.08	11.08	0.06	11.03	0.10	0.858
*Faecalibacterium*	5.79	0.34	5.36	0.24	6.45	0.40	0.065
*Turicibacter*	8.25	0.20	8.01	0.14	8.00	0.24	0.593
*Streptococcus*	5.14	0.47	5.78	0.33	5.22	0.57	0.434
*E. coli*	4.90	0.46	5.78	0.32	5.32	0.55	0.267
*Blautia*	10.78	0.12	10.46	0.08	10.47	0.14	0.089
*Fusobacterium*	8.86	0.18	9.11	0.13	9.37	0.22	0.230
*C. hiranonis*	6.86	0.11	6.86	0.08	6.82	0.13	0.970
*Bifidobacterium*	4.86	0.53	6.12	0.37	6.16	0.63	0.145
*Bacteroides*	5.75 ^a^	0.11	5.93 ^a^	0.15	6.75 ^b^	0.26	0.017
*Lactobacillus*	5.76	0.46	5.30	0.33	5.60	0.56	0.680
Dysbiosis index	−4.26	0.72	−3.05	0.51	−4.01	0.86	0.305
Chao 1 index	74.39	9.25	84.92	6.50	98.23	11.08	0.295
Shannon index	3.65	0.26	3.60	0.18	4.16	0.31	0.276

^a,b^: Differences between age categories are indicated using superscript letters.

**Table 5:** Beta diversity analysis comparing the three age categories: Senior (S), Adult (A), and Junior (J). Unweighted UniFrac, weighted UniFrac, and Bray–Curtis distances are shown.

	S vs. A vs. J		S vs. A		S vs. J		J vs. A	
Indicators	R	*p*-Value	R	*p*-Value	R	*p*-Value	R	*p*-Value
Unweighted UniFrac	0.105	0.036 *	0.073	0.94	0.241	0.008 *	0.053	0.186
Weighted UniFrac	0.053	0.112	0.048	0.173	0.053	0.136	0.069	0.128
Bray–Curtis	0.052	0.148	0.012	0.289	0.098	0.041	0.09	0.114

* *p*-value is significant when <0.05.

**Table 6:** Phylum relative abundance (%) split by age category: Junior (J), Adult (A), and Senior (S).

	Junior		Adult		Senior		J vs. A vs. S	
Phylum	Median	Range	Median	Range	Median	Range	*p*-Value	q-Value
Firmicutes	85.47	66.12–97.50	81.13	63.02–96.19	83.41	56.62–99.7	0.076	0.095
Actinobacteria	8.57 ^a,b^	1.53–29.13	11.32 ^a^	1.50–35.84	6.69 ^b^	0.17–22.1	0.005	0.010
Fusobacteria	2.29	0.17–10.49	2.47	0–20.76	3.45	0–36.85	0.265	0.265
Bacteroidetes	0.10 ^a^	0–3.59	0.35 ^a^	0–10.99	1.63 ^b^	0–18.17	0.035	0.010
Proteobacteria	0.05 ^a^	0–0.68	0.16 ^a,b^	0–9.50	0.31 ^b^	0–2.19	0.006	0.010

^a,b^: Differences between age categories are indicated using superscript letters.

**Table 7:** Results of SCFAs, IgA, and cCP split by age category. Values are expressed as estimated marginal means (emmeans) and standard error (SE). The correlation between the biomarkers and age is expressed as rho.

	Junior		Adult		Senior		J vs. A vs. S	
Indicators ^1^	Emmeans	SE	Emmeans	SE	Emmeans	SE	*p*-Value	Rho
cCP (ng/g)	4.82	0.75	4.14	0.53	2.81	0.90	0.262	−0.075
IgA (mg/g)	12.49	4.14	8.24	2.91	4.96	4.96	0.521	−0.197
SCFA concentrations (μmol/g DM)								
Acetate	145.92 ^a^	34.99	250.29 ^b^	24.61	263.29 ^b^	41.94	0.048	0.358 *
Propionate	11.77	1.89	13.46	1.33	11.67	2.26	0.641	0.196
Butyrate	52.60	11.13	82.07	7.83	61.21	13.35	0.071	0.239
Isobutyrate	7.93	1.56	9.23	1.10	6.14	1.87	0.326	0.059
Isovalerate	11.15	2.32	8.58	1.63	8.12	2.78	0.622	0.066
Valerate	5.07	3.43	12.19	2.41	6.96	4.11	0.180	0.224
Total SCFAs	234.44	46.64	375.85	32.80	357.4	55.90	0.055	0.365 *
SCFA proportions (%)								
Acetate	67.11	2.69	65.41	1.89	72.91	3.22	0.134	0.111
Propionate	4.94 ^a^	0.34	3.65 ^b^	0.24	3.35 ^b^	0.41	0.007	−0.197
Butyrate	19.44	1.95	22.26	1.37	17.45	2.34	0.148	0.078
Isobutyrate	3.04	0.41	2.68	0.29	1.83	0.49	0.195	−0.294
Isovalerate	3.69	0.61	2.54	0.43	2.50	0.73	0.286	−0.213
Valerate	1.78	0.96	3.45	0.67	1.96	1.15	0.250	0.013

^1^ cCP: canine calprotectin; IgA: immunoglobulin A; SCFAs: short-chain fatty acids. ^a,b^: Differences between age categories are indicated using superscript letters. * The rho significant values (*p*-value < 0.05) are marked with an asterisk.

**Table 8:** Results of B9, B12, CRP, and HP split by age category. Values are expressed as estimated marginal means (emmeans) and standard error (SE). The correlation between the biomarkers and age is expressed as rho.

	Junior		Adult		Senior		J vs. A vs. S	
Indicators ^1^	Emmeans	SE	Emmeans	SE	Emmeans	SE	*p*-Value	Rho
B9 (ng/mL)	15.15 ^b^	1.18	13.60 ^b^	0.83	20.37 ^a^	1.42	0.001	0.144
B12 (pg/mL)	652.39	49.85	607.19	35.06	511.99	59.75	0.226	−0.424 *
CRP (μg/mL)	3.67	4.24	5.45	2.98	2.21	5.08	0.827	−0.231
HP (g/L)	1.38	0.48	1.40	0.33	2.32	0.57	0.346	0.426 *

^1^ B9: folate; B12: cobalamin; CRP: C-reactive protein; HP: haptoglobin. ^a,b^: Differences between age categories are indicated using superscript letters. * The rho significant values (*p*-value < 0.05) are marked with an asterisk.

**Table 9:** Results of the behavioral biomarkers split by age category. Estimated marginal means (emmeans) and standard error (SE) of TRP, KYN, KTR, CORT, OT, and TT4 are shown.

	Junior		Adult		Senior		J vs. A vs. S	
Indicators ^1^	Emmeans	SE	Emmeans	SE	Emmeans	SE	*p*-Value	Rho
TRP (μg/mL)	11.29	2.00	11.93	1.37	11.64	2.29	0.965	0.069
KYN (ng/mL)	646.70	101.87	770.39	69.69	957.10	116.37	0.158	0.217
KTR	61.73	15.28	79.85	10.45	97.22	17.46	0.332	0.167
CORT (μg/dL)	0.13	0.02	0.14	0.01	0.15	0.02	0.786	0.061
OT (pg/mL)	2747.32	411.39	1904.73	292.08	1991.45	481.48	0.257	−0.247
TT4 (μg/dL)	1.91 ^a^	0.14	1.42 ^b^	0.09	1.43 ^b^	0.16	0.021	−0.241

^1^ TRP: tryptophan; KYN: kynurenine; KTR: kynurenine/tryptophan ratio; CORT: cortisol; OT: oxytocin; TT4: total thyroxine. ^a,b^: Differences between age categories are indicated using superscript letters.

**Table 10:** Results of the behavioral observations (made in the park and in the kennels) and activity measurements expressed as estimated marginal means (emmeans) and standard error (SE), split by age category. The table only shows behaviors with a relative frequency of more than 10% in the emmeans in at least one age category.

	Junior		Adult		Senior		J vs. A vs. S	
Indicators ^1^	Emmeans	SE	Emmeans	SE	Emmeans	SE	*p*-Value	Rho
Observations in the parks (%)								
P	16.71 ^a^	2.58	2.26 ^b^	1.75	2.48 ^b^	3.01	<0.001	−0.435 *
EX	19.68	6.10	28.77	4.13	38.09	7.13	0.170	−0.346 *
HA	7.81 ^a^	4.51	24.02 ^b^	3.06	20.78 ^b^	5.28	0.018	0.252
Observations in the kennels (%)								
EX	12.64 ^a^	2.72	5.87 ^b^	1.92	1.40 ^b^	3.27	0.013	−0.501 *
HA	6.50	3.22	11.03	2.26	8.11	3.86	0.463	0.020
Activity (bits)								
TAC	1,369,452	197,048	1,123,123	133,524	980,744	230,455	0.424	−0.474 *
DAC	1,246,316	973,358	973,358	117,886	820,826	203,465	0.271	−0.502 *
NAC	123,137	45,711	149,765	30,975	159,918	53,461	0.851	0.074

^1^ P: playing; EX: exploring; HA: high alert; TAC: total activity; DAC: diurnal activity; NAC: nocturnal activity. ^a,b^: Differences between age categories are indicated using superscript letters. * The rho significant values (*p*-value < 0.05) are marked with an asterisk.

## Data Availability

Data access is restricted to protect confidential or/and proprietary information but can be made available upon request, with permission, for the purposes of peer review.

## Supplementary Materials

The following supporting information can be downloaded at https://www.mdpi.com/article/10.3390/ani15020234/s1. Table S1. Microbial family results organized by phylum of the 16s rRNA sequencing analysis split by age category; Table S2. Table showing the correlation between the biomarkers of intestinal health vs. the biomarkers of behavior.
