# Supplementary material for: Age-Related Changes in Gut Health and Behavioral Biomarkers in a Beagle Dog Population
Source: Animals (Basel). 2025 Jan 16;15(2):234. doi: 10.3390/ani15020234 (PMC11758293; doi:10.3390/ani15020234)
Supplement: Supplementary file 1 [file animals-15-00234-s001.zip › animals-3370306-Table S1.pdf]

Table S1. Microbial family results organized by phylum of the 16s rRNA sequencing analysis split by age category.

		Junior		Adult		Senior		J v A v S	
**Phylum**	**Family**	**Median**	**Range**	**Median**	**Range**	**Median**	**Range**	**P value**	**Q value**
Actinobacteria		8.57[a,b]	1.53-29.13	11.32[a]	1.5-35.84	6.69[b]	0.17-22.13	0.005	0.010
	Coriobacteriaceae	8.2	1.22-29.13	5.89	0.72-32.14	5.33	0.17-18.59	0.104	0.164
	Bifidobacteriaceae	0.01[b]	0-0.71	1.08[a]	0-33.43	0.04[a,b]	0-17.45	0.004	0.017
Bacteroidetes		0.1[a]	0-3.59	0.35[a]	0-10.99	1.63[b]	0-18.17	0.004	0.010
	Muribaculaceae	0	0-0.46	0	0-10.83	0.01	0-7.71	0.068	0.125
	Bacteroidaceae	0.04[a]	0-1.36	0.1[a]	0-3.39	0.62[b]	0-9.85	0.004	0.017
	Prevotellaceae	0[a]	0-0.7	0[a]	0-1.06	0.06[b]	0-6.87	0.001	0.009
	[Paraprevotellaceae]	0.05[a,b]	0-1.03	0.04[a]	0-1.11	0.2[b]	0-8.49	0.009	0.026
	Turicibacteraceae	0.66	0-25.76	0.98	0-34.01	1.08	0-33.41	0.728	0.728
Firmicutes		85.47	66.12-97.5	81.13	63.02-96.19	83.41	56.62-99.77	0.076	0.095
	Lachnospiraceae	15.18[b]	6.25-26.19	10.84[a]	1.25-29.46	13.37[a,b]	0.15-34.7	0.012	0.027
	Streptococcaceae	0.21	0-29.4	0.12	0-41.69	0.22	0-64.62	0.668	0.700
	Clostridiaceae	36.97	8.2-62.07	33.84	2.96-65.74	29.55	0.78-58.95	0.547	0.602
	Erysipelotrichaceae	11.42	2.23-24.91	12.13	2.13-83.47	7.9	0.11-52	0.099	0.164

	Lactobacillaceae	0.1	0-67.81	0.61	0-53.67	1.11	0-97.91	0.379	0.439
	Peptococcaceae	0.51[b]	0-1.33	0.2[a]	0-1.84	0.15[a]	0-0.85	0.009	0.026
	Uncl. *ClostridialesI*	0.15[b]	0-0.43	0.05[a]	0-0.34	0.17[b]	0-0.7	0.002	0.014
	Uncl. *ClostridialesII*	0.32	0-0.97	0.15	0-0.83	0.2	0-0.94	0.134	0.197
	Ruminococcaceae	0.24[a,b]	0-3.37	0.25[a]	0-2.34	0.85[b]	0-11.69	0.005	0.017
	Peptostreptococcaceae	0.9	0-11.41	0.18	0-8.06	0.27	0-1.87	0.246	0.319
	Veillonellaceae	0[b]	0-0.2	0.11[a]	0-11.13	0.17[b]	0-10.71	0.000	0.001
Fusobacteria		2.29	0.17-10.49	2.47	0-20.76	3.45	0-36.85	0.265	0.265
	Fusobacteriaceae	2.29	0.17-10.49	2.47	0-20.76	3.45	0-36.85	0.265	0.324
Proteobacteria		0.05[a]	0-0.68	0.16[a,b]	0-9.5	0.31[b]	0-2.19	0.006	0.010
	Enterobacteriaceae	0	0-0.24	0.01	0-9.45	0.01	0-1.59	0.233	0.319
	Alcaligenaceae	0.02[a]	0-0.22	0.04[a,b]	0-1.66	0.1[b]	0-2.13	0.031	0.061
	Succinivibrionaceae	0[a,b]	0-0.58	0[a]	0-1.49	0.03[b]	0-0.56	0.012	0.027
